# Poultry Coccidiosis in and Around Arba Minch, Southern Ethiopia: A Study Based on Coprological and Postmortem Findings

**DOI:** 10.1002/iid3.70499

**Published:** 2026-08-18

**Authors:** Yacob Bekele, Minale Getachew, Mitiku Yohannes, Ephrem Tora

**Affiliations:** ^1^ Arba Minch City Agricultural Department Livestock Office Arba Minch South Ethiopia; ^2^ Department of Veterinary Science, Animal Sciences College of Agricultural Science Arba Minch University Arba Minch South Ethiopia

**Keywords:** coccidiosis, *Eimeria*, lesion, poultry, prevalence, risk factors

## Abstract

**Background and Objectives:**

Ethiopia has a large chicken population, but its economic contribution is limited by disease challenges, especially coccidiosis. Despite coccidiosis's economic and health impacts, there is a notable gap in detailed knowledge regarding the predominant *Eimeria* species, associated intestinal lesions, and distribution across production settings in and around Arba Minch. This lack of comprehensive epidemiological and pathological facts is paramount to develop effective, evidence‐based prevention and control strategies.

**Materials and Methods:**

A cross‐sectional study was conducted between September 2023 and April 2024 in and around Arba Minch town, southern Ethiopia, to estimate coccidiosis prevalence, identify *Eimeria* species, and assess associated risk factors for two exotic chicken breeds (Sasso T44 and Bovans Brown). The study also involved fecal flotation techniques, postmortem examination of chickens' intestines for gross lesions, and mucosal scraping to determine the predilection site of *Eimeria* species and identification of *Eimeria* (*E*.) species.

**Results:**

A total of 1004 examined chickens, 219 (21.81%) harbored different *Eimeria* species. In the current study, statistically significant differences (*p* < 0.05) were observed in the clinical status of the chickens, anticoccidial provision as prophylaxis, litter moisture, and water source. In this study, five species of *Eimeria* were identified: *Eimeria tenella*, *Eimeria necatrix*, *Eimeria maxima*, *Eimeria brunetti*, and *Eimeria acervulina* with proportions of 37.44%, 18.72%, 16.44%, 15.53%, and 11.87%, respectively. *E. tenella* was the predominant species in the study area. Postmortem examination was carried out on 24 birds, out of which 19 (79.17%) were positive for coccidiosis by mucosal scraping examination, and pathological changes/lesions were observed on different parts of the small intestine and cecum.

**Conclusion:**

The present study showed that coccidiosis is an important disease of poultry in the study area and strict sanitary and preventive measures needed to be implemented to reduce the loss due to poultry coccidiosis.

## 1 Introduction

A report by the Food and Agriculture Organization [1] indicates that there are about 20 billion chickens worldwide, with roughly 75% found in developing countries [2]. Poultry farming serves as an important source of income and nutrition for smallholder farmers in developing countries. In most rural communities, women play a central role in managing and sustaining poultry production, contributing significantly to household food security and livelihoods. Beyond its economic importance, poultry is widely recognized across the modern world as the leading source of affordable, high‐quality animal protein, providing essential nutrients that support human health and well‐being [3].

Ethiopia has an outsized poultry population, estimated to be 48.89 million, with indigenous breeds, hybrid chickens, and exotic breed chickens primarily kept in urban and peri‐urban areas, accounting for 81.7%, 10.9%, and 7.4%, respectively [4]. So far, three types of poultry production systems have been identified for managing these poultry populations. Backyard poultry production systems, small‐scale intensive poultry production systems, and large‐scale intensive poultry production systems are examples of poultry production systems in Ethiopia. In all production systems, chickens are raised to produce eggs and meat for income and domestic consumption [5]. Furthermore, 99% of the chicken population is raised using the traditional backyard management system, while 1% is raised using the intensive management system. Traditional production system is characterized by minimum inputs: chicken gather food, receive simple housing like sharing house with their owners at night, and breed naturally which leads to low output from the production [6].

In Ethiopia, poultry production systems are hampered by diseases, predation, malnutrition, improper housing, and poor management system. The most common diseases of chickens in Ethiopia are Newcastle disease, coccidiosis, salmonellosis, chronic respiratory disease, and nutritional deficiency [7]. Mortality following these diseases in chickens is estimated between 20% and 50% but can go as high as 80% during epidemics [8]. Coccidiosis is one of the most common and economically damaging poultry diseases in Ethiopia, consistently highlighted by several studies for its high prevalence and significant impact on chicken production [6, 7]. Currently, poultry production is becoming more intensified which give greater chance to direct contact with their feces in a moist environment from deep litter system in flooring that ensures survival of the oocysts and their transmission from flock to flock [9].

Poultry coccidiosis is caused by single‐celled protozoan parasites of the genus *Eimeria* and has nine species. The most common and pathogenic species that affect poultry is *Eimeria tenella*, resulting in 100% morbidity and high mortality [10]. In Ethiopia, *Eimeria acervulina, Eimeria necatrix, Eimeria maxima*, and *E. tenella* are endemic in all parts of the country and affect mainly young growing birds [11]. Life cycle of all *Eimeria* species involves asexual (shizogony) and sexual phase (gametogony) which form oocyst [12, 13]. Infection occurs by ingestion of sporulated oocyst, followed by the release of sporozoites in the duodenal lumen by mechanical and chemical action. Sporozoites invade the mucosa to start intracellular growth and asexual multiplication with periodic release of merozoites (develop to macro/male and micro/female gametes). Sexual phase occurs, fertilization of macrogamete by microgamete results in the formation of a zygote which is called as an oocyst [14]. The life cycle is rapid and may result in hundreds of thousands or even millions of oocysts produced from a single oocyst within 4–5 days [6].

Poultry coccidiosis distresses birds in both clinical and subclinical forms. The clinical form of the disease is manifested by noticeable signs of mortality, morbidity, diarrhea or bloody feces, dehydration, lowered feed intake, weight loss, paleness, huddled, ruffled feathers, and depression [15]. Chickens affected by coccidia become tired and weak; young birds often have blood in their faces after 4–5 days; later their eyes are closed and their wings hang down, and many birds die [16]. The occurrence of clinical coccidiosis is directly related to the number of sporulated oocysts ingested by a bird at one time, the pathogenicity of *Eimeria* species, the age of the infected chicken, and the management system. Chickens suffering from coccidiosis quickly become less productive and poor performers. Laying hens will experience a reduction in the rate of egg production [17]. Subclinical coccidiosis is characterized by low weight gain and feed conversion efficiency, which are responsible for the greatest proportion of total economic losses [16].

Identification of an oocyst in faces and postmortem assessment of digestive sores are the main strategies for the determination of coccidiosis in chicken [18]. Regardless of its significance of the diseases in Ethiopia, particularly in the study area, no substantial research was done.

As an almost universally significant poultry disease, coccidiosis remains endemic in Ethiopia and continues to threaten productivity and profits [19]. It warrants rigorous investigation and continuous surveillance to effectively mitigate its impact and enhance overall production and productivity [20]. Occurrence of coccidiosis is connected with different variables like host, agent, and environmental interaction [6]. Knowing that poultry coccidiosis is host‐specific and that each *Eimeria* species targets a particular part of the intestine is crucial for developing effective control and prevention measures [21]. More knowledge of the etiology and population dynamics of coccidial infections in commercial poultry production is consequently needed [22]. Poultry is the most important animal species in and around Arba Minch town both for nutritional value they contribute and cash income generation than other animals since they are the main resources especially for poor families. As a poultry farming continues to expand, it is important to regularly estimate the prevalence of coccidiosis, assess the distribution of *Eimeria* species, and related potential factors in the study area.

## 2 Materials and Methods

### 2.1 Description of Study Area

**Figure 1:**
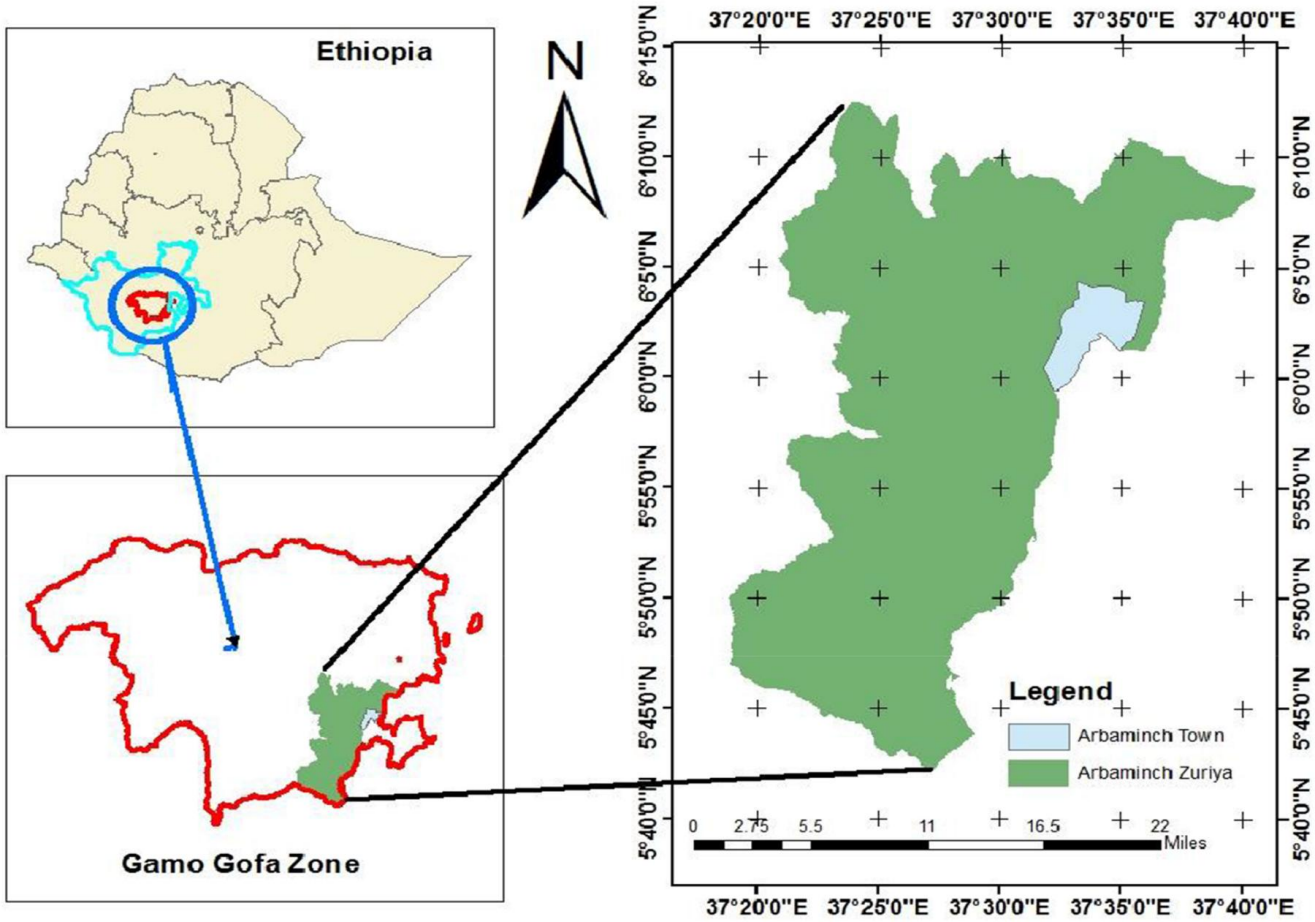
The location of study area on map.

The study was carried out in and around Arba Minch, a town located in Southern Ethiopia (Figure 1). Arba Minch serves as the administrative center of the Gamo Zone. The area is bordered by the Wolayta Zone to the north, the Rift Valley lakes, Lake Abaya and Lake Chamo, to the east, and Segene together with parts of the South Omo Zone to the south. Geographically, the study area is situated approximately 505 km south of Addis Ababa and lies between 5°57′–6°71′ N latitude and 36°37′–37°98′ E longitude. The elevation of the area ranges from 680 to 4207 meters above sea level. The region receives an annual rainfall of about 600–1600 mm, while the mean annual temperature ranges between 10°C and 34°C [4].

Arba Minch Zuria District is located within the well‐known East African Rift Valley and is surrounded by Lake Chamo, Lake Abaya, and Nech Sar National Park. The area is characterized by diverse topographical features, including massifs, plains, steep slopes, and deep gorges formed along several streams, rivers, and lakes. The district, together with other parts of the Gamo Zone, experiences a bimodal rainfall pattern. The short rainy season extends from January to April, while the main rainy season occurs from June to September. Annual rainfall in Arba Minch and the surrounding Zuria district ranges from 800 to 1200 mm, with an average annual temperature of about 26.33°C. The altitude of the district ranges from 1001 to 2500 meters above sea level. According to the 2019 report from the Arba Minch Zuria District and Gamo Zone Livestock and Fishery Resource Office, the district has an estimated livestock population of 101,628 cattle, 27,339 sheep, 42,662 goats, 3204 equines, and 140,050 poultry. In addition, there are 48 poultry farms in Arba Minch and approximately 17 poultry farms in Arba Minch Zuria District, most of which operate under intensive production systems.

### 2.2 Study Population

The study animals were Bovans Brown and Sasso T44 breeds of chickens in poultry farms and owned by local farmers in and around the town. The study chickens were grouped into breeds (Brown Bovans and Sasso T44), and ages classified as young (chicks, young, and adult older than 2 months) [23]. The study chickens are kept under an intensive farming system. The study included kebeles and farms that keep exotic poultry breeds under an intensive production system, covering all age groups. Kebeles with local breeds managed extensively were excluded from the assessment.

### 2.3 Study Design

A cross‐sectional study design was employed from September 2023 to April 2024, to estimate the prevalence of poultry coccidiosis, assess associated risk factors, and examine the pathological lesions in affected chickens within the study area. The study involved randomly selected chickens from intensive production system, where fecal samples were collected and examined for *Eimeria* oocysts to determine prevalence. Concurrently, data on potential risk factors such as age, breed, management system, housing conditions, and hygiene practices were gathered through observation and structured questionnaires. In addition, clinically suspected birds were subjected to postmortem examination to identify and characterize intestinal lesions associated with coccidiosis, thereby linking parasitological findings with pathological manifestations.

### 2.4 Sampling Techniques and Sample Size Determination

Study districts, Arba Minch town and Arba Minch Zuria, were selected purposefully based on the number of chickens on the farms. A two‐stage cluster sampling method was used to select poultry farms from the study area. First, poultry farms/clusters were sampled using simple random sampling. Next, individual chickens from the selected cluster were sampled using a simple random sampling method. Ink was applied to each sampled chicken in order to avoid double sampling. To ensure manageable fieldwork, the number of individual chicks to be sampled per cluster was set at 30 (*n* = 30). Design effect is an adjustment factor used to allow for clustering [24]. The design effect (D) was calculated using the formula *D* = 1 + (*n* − 1) × ICC, where the intra‐cluster correlation coefficient (ICC) was 0.0157. Thus, the design effect was calculated as *D* = 1 + (30 − 1) × 0.0157 = 1.4553. The required number of clusters was then determined using the formula:

$$g=\frac{1.96^{2}\;\times \;D\times \;P\exp\;(1-P\exp)}{nd^{2}}$$

where *Z* = 1.96 for a 95% confidence level, *P*exp = 0.37, *d* = 0.05, and *n* = 30. Substituting these values gave 17.38, which was rounded to 17 clusters. Therefore, the required sample size was initially estimated at 502 chickens, based on 17 clusters with 30 chickens sampled per cluster. To increase the study's statistical power and improve the precision of prevalence estimates, the number of clusters was doubled while maintaining the same average cluster size, resulting in a final sample size of 1004 chickens (34 clusters × 30 chickens per cluster).

The sample size for determining Household for questionnaire survey was obtained using probability proportionate sampling technique [25]:

$$n=\frac{{\;z}^{2}*p(q)}{d^{2}}$$

where *n* represents the required sample size according to Cochran [25] for populations greater than 10,000; *z* is the standard normal deviation corresponding to a 95% confidence level (1.96); *p* is the estimated proportion of the population included in the sample, taken as 0.1 (10%); *q* is 1 − *p*, which equals 0.9 (90%); and *d* represents the desired level of precision set at 0.05 (5%). Based on this formula, a total of 138 respondents were selected from the districts for the study.

### 2.5 Data Collection

#### 2.5.1 Questionnaire

Semi‐structured questionnaire was used to collect demographic and basic information about the production system and the management practice in the study area. A total of 138 households were used for semi‐structured questionnaire surveys and were proportionally allocated for each study site (Survey).

#### 2.5.2 Parasitological Examination

Fecal samples from each bird were collected with a spatula from freshly voided feces. Fecal samples were collected in sampling universal bottles, identified appropriately, and transported to Arba Minch University College of Agricultural Sciences, veterinary laboratory for analysis. Before microscopic observation, floatation techniques were performed to concentrate the oocysts in order to increase the sensitivity of the examination. Sodium chloride solution was used as a floating medium. The diagnosis of the oocysts in the feces was made using a 10x optical lens of the microscope.

#### 2.5.3 Euthanasia

The study birds were humanely euthanized using cervical dislocation, a quick and widely accepted method that reduces pain and distress when carried out by trained personnel [26]. The procedure involved gently restraining the bird and then swiftly dislocating the cervical vertebrae from the skull to ensure rapid death [27, 28].

#### 2.5.4 Posmortem Examination

After confirming death, a necropsy was carried out to examine the predilection sites of *Eimeria* and the associated pathological lesions. The bird was positioned dorsally, and the abdominal cavity was opened using sterile instruments. The gastrointestinal tract, particularly the small intestine and caeca, was carefully examined for *Eimeria* oocysts and related changes [29, 30]. The intestinal portions were divided into four sections: the upper part (duodenum and jejunum), the middle part (ileum), the lower part (distal ileum and rectum), and cecal pouches. Intestinal contents and mucosal surfaces were also inspected, with microscopic confirmation. The intestinal wall of different sites was examined for thickening, petechiae, coagulative necrosis, reddening, whitish spots, cecal cores, or bleeding. Then, the mucosal scrapings were taken to examine the protozoan developmental stage along with lesions by microscopic examination [29, 31]. Finally, the observed gross pathological lesions were recorded.

#### 2.5.5 Identification of Species of Eimeria

Species of *Eimeria* were identified by combination of microscopic features of oocyst morphology (shape, size, sporulation time, and color of the oocysts), the predilection site of *Eimeria* in the gut, and the nature of gross lesions by postmortem examination.

### 2.6 Data Management and Analysis

All data were recorded in a predesigned format, and entered into Microsoft Excel, cleaned, and coded prior to analysis. Analyses were conducted in R version 4.5.2 [32] using the RStudio integrated development environment version 2026.01.1‐403. Analyzed data were presented using tables, percentages, and odds ratios. Binary logistic regression was performed to identify factors independently associated with the outcome variable. Initially, univariate logistic regression analyses were conducted for each explanatory variable. Variables with a *p* < 0.25 in the bivariable analysis, as well as variables considered clinically based on previous literature, were entered into the multivariable logistic regression model. Multicollinearity among independent variables was assessed before model fitting using variance inflation factors (VIFs). Variables with gamma values ranging between −0.6 and +0.6 were retained and included in the multivariable logistic regression model. Model goodness‐of‐fit was evaluated using the Hosmer–Lemeshow test. Adjusted odds ratios (AORs) with 95% confidence intervals (95% CIs) and corresponding *p* values were reported. Throughout the analysis, statistical significance was set at *p* < 0.05.

## 3 Results

### 3.1 Prevalence of Coccidiosis

The result of the present study illustrates that poultry coccidiosis is endemic in and around Arba Minch town, South Ethiopia with an overall prevalence of 21.81% (219/1004). Out of 314 chickens examined from Arba Minch town and 690 from Arba Minch Zuria District, 68 (21.66%) and 151 (21.88%) were found infected by different *Eimeria* species, respectively (Table 1).

**Table 1:** Prevalence of coccidiosis in the two districts of the study areas.

Administrative area	Kebeles	No. examined	No. positive	Prevalence (%)
Arba Minch town	Doysa	46	10	21.74%
	Bere	62	12	19.35%
	Wuha Minch	74	15	20.27%
	Gurba	40	9	22.50%
	Edget Berr	34	8	23.53%
	Limat Woze	58	14	24.14%
	Total	314	68	21.66%
Arba Minch Zuria	Genta Bonke	126	28	22.22%
	Genta Meche	162	37	22.84%
	Zeyse Wozeka	202	45	22.28%
	Shelle mela	66	15	22.73%
	Chano mile	50	11	22%
	Lante	84	15	17.86%
	Total	690	151	21.88%
Total in districts		1004	219	21.81%

In this study, the prevalence of coccidiosis was found to be 20.57% in chicks, 23.53% in growers, and 20.98% in adult chickens. When comparing breeds, the infection rate was 20.47% in Brown Bovans and 22.99% in Sasso T44 chickens (Table 2). Moreover, coccidiosis was more common among chickens that appeared sick (29.48%) compared to those that appeared healthy (18.07%), indicating a strong association between the disease and visible illness symptoms.

**Table 2:** Overall prevalence of poultry coccidiosis in the study area.

Variables	Category	No. examined	No. positive	Prevalence (%)
Age	Chick (< 6 wks)	282	58	20.57%
	Grower (7−15 wks)	374	88	23.53%
	Adult (> 16 wks)	348	73	20.98%
Sex	Male	379	80	21.11%
	Female	625	139	22.24%
Breed	Brown Bovans	469	96	20.47%
	Sasso	535	123	22.99%
Clinical status	Clinical	329	97	29.48%
	Subclinical	675	122	18.07%
Body conditions	Poor	122	30	24.59%
	Moderate	320	67	20.94%
	Good	562	122	21.71%
House type	Floor	706	160	22.66%
	Cage	298	59	19.80%
Litter moisture	Wet	217	66	25.96%
	Dry	489	94	19.44%
Feed and water sanitation	Poor	731	163	22.30%
	Good	273	56	20.51%
Water source	Tap water	241	175	18.26%
	River water	763	44	22.94%
Feed source	Commercial feed	647	127	19.63%
	Home‐mixed feed	357	92	25.77%
Anticoccidial provision	Provided	923	180	19.50%
	Not provided	81	39	48.15%

### 3.2 Risk Factors Associated With Prevalence of Poultry Coccidiosis

All risk factors were assessed for the presence of statistically significant association between putative factors and the prevalence of poultry coccidiosis using univariable logistic regression analysis. Among the potential risk factors, three variables were found to be statistically (*p* < 0.05) associated with the prevalence of poultry coccidiosis in the analysis of univariate logistic regression (Table 3).

**Table 3:** Univariable logistic regression of risk factors with coccidiosis in the study area.

Variables	Category	No. examined	No. positive	Odd ratio	95% CI	*p*
Age	Adult (> 16 wks)	348	73	Ref		
	Chick (< 6 wks)	282	58	1.00	0.66–1.53	0.980
	Grower (715 wks)	374	88	1.20	0.81–1.79	0.360
Sex	Male	379	80	Ref		
	Female	625	139	1.05	0.75–1.47	0.760
Breed	Brown Bovans	469	96	Ref		
	Sasso	535	123	1.11	0.81–1.53	0.510
Clinical status	Subclinical	675	122	Ref		
	Clinical	329	97	1.47	1.05–2.07	0.026
Body conditions	Good	562	122	Ref		
	Poor	122	30	1.09	0.67–1.75	0.73
	Moderate	320	67	0.86	0.52–1.44	0.572
House type	Cage	298	59	Ref		
	Floor	706	160	1.23	0.87–1.75	0.24
Litter moisture	Dry	489	94	Ref		
	Wet	217	66	0.46	0.11–1.95	0.294
Feed and water sanitation	Good	273	56	Ref		
	Poor	731	163	1.09	0.77–1.56	0.62
Water source	Tap water	241	175	Ref		
	River water	763	44	1.49	1.02–2.20	0.040
Feed source	Commercial feed	647	127	Ref		
	Home‐mixed feed	357	92	.65	0.16–2.77	0.566
Anticoccidial	Provided	923	180	Ref		
	Not provided	81	39	3.12	1.87–5.20	0.000

The final logistic regression model revealed that clinical status, water source, litter moisture, anticoccidial provision remained significant predictors of poultry coccidiosis in farms (*p* < 0.05) (Table 4). The fitness of the model for the data was also checked. The Hosmer–Lemeshow goodness‐of‐fit test suggested that the model fit the data (*χ* ^2^ = 7.79; *p* = 0.45).

**Table 4:** Multivariable logistic regression analysis on different risk factors.

Risk factors	Category	OR	95% CI	*p*
Clinical status	Subclinical	Ref		
	Clinical	1.49	1.06–2.09	0.022
Litter moisture	Wet	Ref		
	Dry	1.42	1.04–1.94	0.027
Water source	Tap water	Ref		
	River water	1.97	1.58–2.69	0.046
Anticoccidial	Provided	Ref		
	Not provided	3.11	1.87–5.16	0.000

The odds of having coccidia in clinically sick birds was 49% more likely to harbor coccidia than the subclinical cases (OR = 1.49; *p* = 0.022). The odds of chickens housed in the wet litter system were having coccidia 42% more likely than the dry litter system with statistically significant difference (OR = 1.42; *p* < 0.027) (Table 4). The odds of getting coccidiosis for the chickens drinking river water had 1.97 times (97% higher) odds of experiencing the outcome compared with those using tap water.

### 3.3 Postmortem Examination

**Figure 2:**
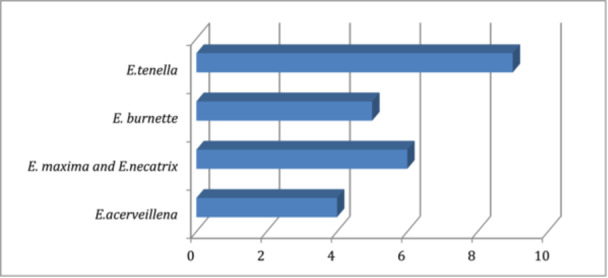
Species‐wise prevalence of *Eimeria* identified by postmortem examination.

During postmortem examination, specific *Eimeria* species were identified based on characteristic lesions that varied in severity and distribution along the intestinal tract according to the predilection sites of each species (Table 5). Of the 24 birds examined, 19 (79.17%) were found to have coccidiosis, as confirmed by mucosal scrapings and visible lesions in the intestines and caeca. *E. tenella* is one of the most widespread species that induced hemorrhage, clotted blood, and cecal cores in the cecum (Figure 2). *E. necatrix* and *E. maxima* usually shared similar intestinal lesions. Lesions in *E. necatrix* were more severe with bleeding and whitish plaques seen in the middle intestine on both sides from yolk sac diverticulums (Table 5).

**Table 5:** Number of cases with *Eimeria* species‐specific lesions identified at predilection sites during postmortem examination.

Site affected	Nature of gross lesion	Species identified	No. of cases with lesion
Duodenum	White lesions and the mucosa sometimes show hemorrhagic appearance	*E*. *acerveillena*	4
Jejunum	Petechiae, the jejunum thickened and ballooned with red pinpoint	*E*. *maxima* and *E*. *necatrix*	6
Ileum	Thin intestinal wall and hemorrhages	*E. brunetti*	5
Cecum	Thickened and ballooned, its content mixed with blood	*E*. *tenella*	9

### 3.4 Identification of Species of *Eimeria*

Five *Emerian* species, namely *E. tenella*, *E. necatricx*, *E. maxima*, *Eimeria brunetti*, and *E. acervulina*, were identified. *E. tenella* was the most prevalent (37.44%), while the other four species occurred at lower frequencies*: E. necatrix* (18.72%), *E. maxima* (16.44%), *E. brunetti* (15.53%), and *E. acervulina* (11.87%) (Figure 2 and Table 5).

### 3.5 Questionnaire Survey

#### 3.5.1 Biography of Respondents

Of the 138 poultry owners, 69 from Arba Minch city administration and 69 from Arba Minch Zuria district were involved in the survey to investigate the awareness about the poultry rearing practice. In total, 61(44.2%) respondents were male and 77(55.8%) were female respondents (Table 6).

**Table 6:** Respondent's bibliography.

Variable	Category	Respondents	Distributions
Sex	Male	61	44.2%
	Female	77	55.8%
Age	Young	55	39.9%
	Adult	83	60.1%
Poultry keeping experience	< 1 year	2	1.45%
	1–5 years	54	39.13%
	> 5 years	82	59.4%
Awareness about coccidiosis	No	98	71.02
	Yes	40	28.98

#### 3.5.2 Production, Disease Problems, and Awareness About Coccidiosis

The survey indicated that the majority (56.52%) of the respondents kept the Bovans brown breed, 33.33% kept the Sasso T44, and 10.14% of farmers reared another breed (Table 7). Poultry farmers sourced their chicks primarily from private hatcheries. It also revealed that both farms and household poultry keepers use a range of antimicrobial and antiprotozoal drugs for treatment. The most commonly used medicinal products were oxytetracycline, amprolium, and vitamin supplements, with oxytetracycline being used by the majority of respondents (63.04%). However, antibiotic use was largely not based on veterinary prescriptions, but rather on the farmers' own decisions.

**Table 7:** Production, disease problems, and awareness about coccidiosis in the study area.

Variables	Categories	No. of respondents	Distributions
Breed of chicks reared	Bovans brown	78	56.52%
	Saso	46	33.33%
	Others	14	10.14%
Major challenges facing farms	Disease	103	74.64%
	environmental stress	12	8.69%
	management related	23	16.67%
Major disease problems	Lice	24	17.39%
	Fowl pox	56	40.58%
	Newcastle	89	64.49%
	Coccidiosis	61	44.20%
	Worms	23	16.66%
Vaccination status in farms	Yes	111	80.43%
	No	27	19.56%
Type of treatment used	Oxytetracycline	87	63.04%
	Sulfonamides	76	55.07%
	Amprolium	23	16.66%
	Vitamins	34	24.64%
Awareness about control of coccidiosis	No	98	71.02%
	Yes	40	28.98%
Awareness of coccidiosis predisposing factors	Water source	56	40.58%
	Feed source	66	47.83%
	Market place	22	15.94%
	Working equipment	52	37.68%
Antibiotic use	Veterinarian prescription	53	37.41%
	Owner decision	85	62.59%

## 4 Discussions

A survey of birds in and around Arba Minch town, Southern Ethiopia, found that poultry coccidiosis is widespread, affecting 21.81% (219 of 1004). The observed pathological lesions were consistent with the characteristic intestinal predilection sites and pathogenic effects of different *Eimeria* species. Inflammation, hemorrhages, and tissue damage in the intestines and caeca of affected birds pose a notable challenge to local poultry production. In addition to confirming *Eimeria* infections, these findings demonstrate how the disease can affect birds' health, growth, and productivity. As a result of the relatively high prevalence of the disease, it is imperative to implement effective control measures, such as better farm management, strengthened biosecurity, and the use of anticoccidial treatments, to minimize economic losses.

The study disclosed that poultry coccidiosis is endemic in and around Arba Minch, with a prevalence of 21.81%. This result is consistent with findings reported in Ambo by Oljira et al. [33] and in the Arsi Tiyo district by Ketema and Fasil [34], from Alage, a veterinary college, where they documented a prevalence of 20.57% and 19.5%, respectively. The present study's prevalence of poultry coccidiosis is lower than the findings of Wondimu et al. [6], who reported 42.2% in an intensive farming system in Gondar, Ethiopia, and Asfaw et al. [7], who estimated a 37% pooled prevalence across Ethiopia. It is also considerably lower than the reports from Nigeria [35], which documented prevalence of 52.9% in young chickens and 36.6% in adults. The possible reasons for the varying prevalence of coccidiosis in the current study might be ascribed mainly to the application of preventative precautions, which are basically biosecurity and the use of anticoccidial drugs given at early stages of age.

The present finding shows a comparable prevalence of coccidiosis among chicks (20.57%), growers (23.53%), and adults (20.98%) with no statistically significant difference (*p* > 0.05). It is similarly supported by epidemiological studies indicating that age may not always be a determinant risk factor under certain management conditions [17, 36]. Although coccidiosis is often considered more common in young birds due to their immature immunity, all age groups may remain susceptible when exposed to similar levels of infective oocysts in the environment [37]. In well‐established farm settings, repeated exposure could lead to partial immunity in older birds, while continuous environmental contamination maintains infection pressure across all age categories, thereby equalizing prevalence rates. Similar observations of nonsignificant differences have been reported in cross‐sectional studies where uniform management practices, housing conditions, and biosecurity measures minimize variation among groups [17]. Statistically, the absence of significance (*p* > 0.05) implies that the opportunity for the chickens to pick up the numbers of sporulated oocysts might be equal in all age categories. This may also be influenced by factors like similar production systems, overlapping confidence intervals, and low effect sizes between groups, which may reduce the power to detect differences.

The present study demonstrated that the prevalence of coccidiosis was 20.47% in Brown Bovans and 22.99% in Sasso T44 chickens, with no statistically significant difference between the two breeds (*p* > 0.05). This finding is consistent with epidemiological studies indicating that although genetics may influence susceptibility, management factors in field conditions are often more important determinants [15, 35, 38]. For instance, this study is in line with the finding of Al‐Gawad et al. [39] in Egypt and Alemayehu et al. [40] in Ethiopia, who recorded 21.24% and 23.1%, respectively. The similar prevalence observed in this sample may be due to management and environmental exposure outweighing breed differences [6, 41]. However, it is well known that different breeds of chicken may exhibit different degrees of “susceptibility” or “resistance” to *Coccidia*, including tolerance to infection and speed of recovery from pathological consequences [42]. Genetic susceptibility exists, but in real farm settings it is often subtle compared to the effects of infection pressure [43]. Consequently, both exotic and improved breeds may exhibit comparable levels of infection when exposed to similar risk conditions, indicating that management‐related factors are more critical determinants of coccidiosis prevalence than breed differences alone [44].

The present study demonstrated a significantly higher prevalence of coccidiosis in apparently sick chickens (29.48%) than in apparently healthy ones (18.07%), a finding that is well supported by previous epidemiological and clinical studies on poultry coccidiosis [31]. The literature consistently indicates that clinical disease is associated with heavy *Eimeria* infection, leading to intestinal epithelial damage, hemorrhage, malabsorption, and visible signs such as diarrhea, depression, and poor performance [15]. Taylor et al. [13] found that birds showing clinical illness are more likely to harbor high oocyst burdens and active lesions, increasing the likelihood of detecting infections. The low parasite loads and partial immune responses developed after prior exposure are often characteristics of apparently healthy chickens, resulting in reduced oocyst shedding and minimal or no visible symptoms [45]. It has been documented that clinical versus subclinical manifestations of coccidiosis depend on a variety of factors, including the amount of infection, the type of environment contaminated, the immune system's ability to fight, and the level of stress [15, 42]. High exposure to sporulated oocysts and compromised host immunity typically result in overt disease, whereas lower exposure levels may lead to inapparent infections [46]. Therefore, significantly higher prevalence in clinically sick birds reflects the well‐established link between infection intensity, intestinal damage, and clinical disease expression.

The highest prevalence of coccidiosis was observed in chickens with poor body condition (24.59%) compared to those with medium (20.94%) and good body condition (21.71%). Similarly, a study conducted in intensive poultry farming in Gondar, Ethiopia, reported a significantly higher prevalence of coccidiosis in chickens with poor body condition (72.6%) compared to medium (36.1%) and good (30.5%) body condition, with statistical significance (*p* < 0.05) [6]. This suggests that birds with poor nutrition or compromised body condition are more immunosuppressed, reducing their ability to mount effective immune responses against *coccidial* infection and consequently increasing their susceptibility and oocyst shedding [47]. Studies disclose that *Eimeria* infection damages the intestinal epithelium, leading to impaired nutrient absorption, reduced feeding efficiency, and weight loss, all of which directly contribute to poor body condition [37, 38]. In contrast, well‐conditioned birds generally have better nutritional reserves and stronger immune competence, enabling partial resistance or tolerance to infection, which limit parasite multiplication and clinical expression. Although good body condition does not fully prevent infection, it is often associated with lower parasite burden and reduced disease severity due to improved resilience [48]. This finding may reflect the bidirectional relationship between coccidiosis and body condition, whereby coccidial infection impairs nutrient absorption and contributes to poor body condition, while animals in poor nutritional condition are more susceptible to higher parasite burdens [13].

A higher proportion of coccidiosis was observed in chickens managed under the floor system (22.66%) compared to those in the cage system (19.80%), though the difference was not statistically significant. This trend is consistent with findings reported by Gebretensae et al. [49] who also noted a higher prevalence in floor‐managed birds. The increased occurrence in floor systems may be attributed to greater exposure to sporulated *Eimeria* oocysts present in litter. As highlighted by Chanie et al. [50], poor management practices such as overcrowding, leaking drinkers, and fecal accumulation further exacerbate the problem by creating warm and moist conditions that favor oocyst survival, sporulation, and transmission. The slightly higher proportion of coccidiosis in floor‐reared chickens than in cage systems can be explained by biological and environmental factors, despite the lack of statistical significance. In floor systems, chickens are constantly in contact with litter that easily becomes contaminated with feces, is the main source of infective *Eimeria* oocysts. This frequent exposure increases the chance of ingesting sporulated oocysts and raises the risk of infection. In contrast, cage systems keep birds separated from their droppings, limiting contact and breaking the fecal–oral transmission cycle.

The odds of getting coccidiosis for the chickens that were drinking river water is 97% more likely than the chickens that were drinking tap water. Similarly, Chalchisa and Deressa [51] reported that chickens drinking river water had approximately 1.71 times higher odds of coccidiosis than chickens drinking tap water. This difference can be explained by the high likelihood of river water contamination with fecal materials harboring infective *Eimeria* oocysts. As noted by Yoo et al. [52], the accumulation of feces and subsequent contamination of water sources increase the environmental load of oocysts, thereby enhancing infection risk. This finding also agrees with reports by Wondimu et al. [6] and Graat et al. [53] noted that drinking water source is a key risk factor, emphasizing that the type of water consumed significantly influences the likelihood of coccidiosis. According to Amaral [54] drinking water plays an important role in the transmission of protozoan diseases that are among the most common poultry diseases. Accumulation of feces and contamination of feed and water as in the case of river water by fecal materials increases the number of *Eimeria* species oocysts [55]. The disease is spread through the consumption of contaminated food and water and symptoms include diarrhea with mucus and blood [50, 52].

*Eimeria* species were identified by postmortem examination and by their lesion characteristics and sites of occurrence, with 19 out of 24 birds (79.17%) testing positive for coccidiosis from mucosal scrapings and intestinal and cecal lesions. *E. tenella* is one of the most widespread species that induced hemorrhage, clotted blood, and cecal cores in the cecum. *E. necatrix* and *E. maxima* infections generally produced similar intestinal lesions. However, lesions caused by *E. necatrix* were comparatively more severe and characterized by hemorrhages and the presence of whitish plaques in the middle portion of the intestine, particularly on both sides of the yolk sac diverticulum [56]. *E. acervulina* infection lesions were mainly observed in the duodenal loop and were characterized by mucoid exudates within the intestinal contents, along with distinct white spots that were typically visible from the serosal surface of the intestine [57]. *E. brunetti* infection was detected in the lower part of the intestine and was characterized by small petechial hemorrhages, which were more clearly visible on the serosal surface than on the mucosal surface [58]. The gross pathological findings observed for each species were generally consistent with previous studies [22, 59, 60, 61]. Lesions caused by *E. maxima* and *E. necatrix* infection can sometimes be mistaken for, especially when they occur together with and are complicated by necrotic enteritis [42, 61].

The current study identified five *Eimeria* species, namely *E. tenella*, *E. necatrix*, *E. maxima*, *E. brunetti*, and *E. acervulina*. *E. tenella* occurred most frequently, with a prevalence of 37.44%, whereas the remaining four *Eimeria* species identified were *E. necatrix*, *E. maxima*, *E. brunetti*, and *E. acervulina*, which were lower and accounted for 18.72%, 16.44%, 15.53%, and 11.87%, respectively. This finding is consistent with previous reports from Ethiopia, which documented four species of *Eimeria* infection in poultry, with the exception of *E. maxima* infection [11, 62]. Similarly, studies from different countries, including Nigeria [35], Iran [63], and Pakistan [64], also reported the presence of four *Eimeria* infections in poultry species, although *E. brunetti* infection was not detected in some of these studies. These suggest that *Eimeria* infection in poultry species is widely distributed across different geographical regions. The observed infection prevalence in the chickens suggests that oocysts are being maintained both within the farm and in the surrounding environment. It also indicates possible shortcomings in hygiene practices, particularly inadequate cleaning and disinfection of poultry houses. *E. acervulina* infection was reported as the predominant species in Ethiopia by Ashenafi et al. [62], while studies in Iran by Nematollahi et al. [63] also identified it as the most common species. In contrast, Lobago et al. [29] reported *E. brunetti* infection as the dominant species in Ethiopia. This discrepancy may be due to differences in farm sanitation, use of anticoccidial drugs in feed or water, or variations in chicken breeds.

The present study shows that 74.64% of respondents identified the disease as a major production constraint, and ranked Newcastle disease, coccidiosis, fowl pox, ectoparasites, and helminths as the most important diseases. A participatory and survey‐based study has consistently shown that poultry diseases are the primary bottleneck to its productivity, with a large proportion of farmers reporting frequent disease occurrence in their flocks [8]. In particular, Newcastle disease is widely recognized as the leading cause of mortality and economic loss in village chickens, while coccidiosis remains among the most prevalent endemic diseases. The relatively high proportion of respondents reporting disease constraints in the current study is in agreement with national‐level evidence indicating that infectious and parasitic diseases are widespread and poorly controlled in Ethiopian poultry systems [7]. Among the major diseases surveyed in this study, coccidiosis ranked second (44.20%) because of management‐related factors like poor hygiene, feces accumulation, and extensive floor systems, which make *Eimeria* oocysts more likely to survive and transmit. Indeed, coccidiosis is reported to be highly prevalent in floor production systems where biosecurity is minimal and veterinary services are limited [65]. The high level of reported disease burden could be linked to the low awareness observed among respondents (71.02%), as lack of knowledge on disease transmission and control has been widely documented as a key factor exacerbating poultry health problems, with a study reporting over 90% of farmers lacking adequate disease management awareness [66].

The survey revealed that the farms and chicken‐keeping households use different antimicrobial and antiprotozoal drugs to treat their poultry. The commonly used drugs were oxytetracycline, amprolium, and vitamin supplements. Among these, oxytetracycline was used by the majority of the respondents (63.04%). However, most antibiotic use is not guided by veterinary prescription, as farmers administer the drugs themselves, indicating irrational use in the study area. The present finding is supported by several epidemiological studies in Ethiopia and other developing countries, which consistently report widespread and often unsupervised use of antimicrobials in poultry production. For instance, a study in central Ethiopia has shown that oxytetracycline is the most used drug in poultry farms, with usage of 76%–83%, reflecting its broad availability, affordability, and perceived effectiveness [67]. Similarly, surveys indicate that over 94% of poultry farmers use antibiotics, frequently for both treatment and prevention, often administered without proper diagnosis [68]. Moreover, a large proportion of livestock owners lack awareness of appropriate antimicrobial use, with only about 14% understanding antimicrobial resistance and many relying on self‐prescription or informal drug vendors [69]. These findings align with the current result, where oxytetracycline was predominantly used and antibiotics were largely administered without veterinary guidance, indicating irrational drug use.

This study had several limitations that should be considered when interpreting the findings. First, the study was conducted in and around Arba Minch, and therefore the findings may not be directly generalizable to poultry populations in other regions of Ethiopia with different management systems, environmental conditions, and production practices. Second, the financial resources required to purchase study animals for postmortem examination limited the number of birds that could be included in the study. Consequently, the postmortem sample size may have reduced the statistical power to detect less common lesions or species‐specific associations. Third, coprological examination provides evidence of coccidian oocyst shedding but does not necessarily indicate active clinical disease or distinguish the pathogenic significance of different *Eimeria* species. Although postmortem examination and lesion assessment provided additional information, lesion‐based identification of *Eimeria* species may also be subject to diagnostic limitations. Furthermore, the study did not employ molecular techniques to confirm species identification and characterize circulating *Eimeria* strains.

## 5 Conclusions

This study demonstrates that poultry coccidiosis is an endemic and economically important disease in and around Arba Minch, with a considerable prevalence and clear pathological impacts on affected birds. Coccidiosis is widely distributed across different age groups and breeds, suggesting that host‐related factors play a more critical role. Higher occurrence in clinically sick and poorly conditioned birds, as well as in floor management systems and among chickens exposed to contaminated water sources, indicates the importance of hygiene, nutrition, and biosecurity in disease transmission. The identification of multiple *Eimeria* species further confirms continuous environmental contamination and sustained infection pressure within poultry production systems. Moreover, the study underscores that poultry diseases, particularly Newcastle disease and coccidiosis, remain the major constraints to production, compounded by poor management practices and low farmer awareness regarding disease control. The widespread and unsupervised use of drugs such as oxytetracycline and amprolium further reflects gaps in veterinary service delivery and contributes to irrational drug use, posing risks of antimicrobial resistance and food safety concerns. Overall, the study highlights the urgent need for integrated control strategies, including improved farm management, strengthened biosecurity measures, enhanced farmer education, and better access to veterinary services to reduce the burden of coccidiosis and improve poultry productivity in the study area.

## Author Contributions

**Yacob Bekele:** conceptualization, funding acquisition, methodology, investigation, data curation, formal analysis, writing – original draft, review, and editing. **Minale Getachew:** supervision, conceptualization, investigation, data curation, formal analysis, writing – original draft, review, and editing. **Mitiku Yohannes:** questionnaire administration, methodology, investigation, formal analysis, writing – original draft, review, and editing. **Ephrem Tora:** funding acquisition, methodology, investigation, data curation, formal analysis, writing – original draft, review.

## Ethics Statement

All studies involving human participants for questionnaire, and animal samples were performed in accordance with the principles of the Declaration of Helsinki. Ethical approval was obtained from the Ethical Research Committee of the Arba Minch University approved this study, with the reference number AMU/AREC/14/2017.

## Consent

Informed verbal consent was obtained from all participants prior to their inclusion and participation in the study.

## Conflicts of Interest

The authors declare no conflicts of interest.

## Data Availability

All data related to the manuscript are available from the corresponding author upon a reasonable request.
